# A new method for detecting deception in Event Related Potentials using individual-specific weight templates

**DOI:** 10.1186/1471-2202-14-S1-P34

**Published:** 2013-07-08

**Authors:** Abdulmajeed Alsufyani, Alexia Zoumpoulaki, Marco Filetti, Howard Bowman

**Affiliations:** 1 Centre for Cognitive Neuroscience and Cognitive Systems (CCNCS), School of Computing, University of Kent, Canterbury, Kent, CT2 7NF, UK; 2 Department of computer science, Taif University, Taif, 21974, Saudi Arabia

## 

A new method called the weight template (WT) is proposed for classifying Event related potentials (ERPs) into deceiving and non-deceiving. In this study, EEG data from two P300-based lie detection experiments were analyzed to demonstrate the efficiency of the WT method in detecting deception. A comparison was made with a common method used to measure P300 presence, called Peak-to-Peak, which is believed to be more accurate than other methods in measuring P300 amplitudes [1,2]. One experiment consisted of presenting participants with birth date stimuli and 12 participants were instructed to lie about their own birthday. The other experiment consisted of 15 participants who were instructed to lie about their first names [3]. Using simulated EEG data [4], Receiver Operating Characteristic (ROC) curves were also generated to examine the efficiency of the proposed method in detecting deception in low signal-to-noise ERPs.

Typically, P300-based lie detection systems employ the P300 component to detect concealed information. They present three stimulus types: Probes (**P**), which represent concealed information or crime details and can be recognized only by the guilty person; Irrelevants (**I**), which are frequent and task (crime)-irrelevant, and Targets (**T**), which are irrelevant items, but participants are asked to do a task whenever they see a Target. For practical lie detection, the key comparison is between Probe and Irrelevant ERPs, since, for the *non*deceiver, the former would be an Irrelevant. Importantly, the Probe for a deceiver typically generates a P300 ERP component, which is absent for the Irrelevant. The principle underlying the WT method is that as the Target stimulus is task-relevant, it will evoke a robust P300 pattern for each subject, which we hypothesize is characteristic in form and polarity of that individual's P300. Accordingly, this ***T ***ERP can serve as an individual-specific template, with which to search for the Probe P300. Specifically, the difference between ***T ***and ***I ***ERPs was used as a template (i.e. effectively as a kernel) and this template was applied to ***P ***and ***I ***ERPs.

Using such a template, with some pre-processing steps, we found that the WT achieved significantly better detection performance in comparison to Peak-to-Peak. In the names lie detection, the WT was able to detect deception for 93% in the guilty group compared with 80% by Peak-to peak. The false alarm rates using WT and Peak-to-Peak were 2% and 8% respectively. In the birthdays lie detection, hit rates were 50% using WT and 33% using Peak-to-Peak. The false alarm rates of both methods were 5%. ROC curve analysis also showed that in ERPs with high signal-to-noise ratio (SNR), both methods could detect deception successfully and almost equally. However, the WT performed better in ERPs with low SNR. We thus conclude that the WT is simple and very effective for detecting deception, even in ERPs with low SNR.
